# In this month’s Bulletin

**DOI:** 10.2471/BLT.26.000826

**Published:** 2026-08-01

In the editorial section, Tara Ballav Adhikari et al. (526) introduce WHO’ s treatment guidance for asthma and chronic obstructive pulmonary disease. Flavio Salio et al. (527) call for research on preparation of the health workforce needed for emergency response. 

## Brazil, Canada, India, Malawi

### How to administer the Early Childhood Development Index 2030 

Nicholas Kuzik et al. (530–539) compare telephone to in-person interviews.

## China 

### Better care for people with diabetes 

Wenxi Tang et al. (566–572) describe a public−private management model.

## Madagascar 

### Where are children missing vaccinations?

Nadine Muller et al. (560–565) engage community health workers in a search for zero-dose children.

## Saudi Arabia 

### Digital epidemiology 

Haytham A Sheerah et al. (573–578) make the case for integrated surveillance.

## Global

### Trade-offs in tuberculosis control

Sandip Mandal et al. (540–550) model the impact of different policies.

### Medicines for drug-resistant bacterial infections in children

Phoebe CM Williams et al. (551–559) argue for expedited research and approval.

### Measurement of access to medicines

Iris R Joosse et al. (579–581) debate changes to an indicator.

### Palliative care in humanitarian responses

Hanna Kaade et al. (582–584) request better integration.

